# Supplementary material for: Anti-Human PD-L1 Nanobody for Immuno-PET Imaging: Validation of a Conjugation Strategy for Clinical Translation
Source: Biomolecules. 2020 Sep 29;10(10):1388. doi: 10.3390/biom10101388 (PMC7599876; doi:10.3390/biom10101388)
Supplement: Supplementary file 1 [file biomolecules-10-01388-s001.pdf]

Supplementary Information

1. Synthesis of GGGYK-NHCS-Bn-NOTA

1.1. Solid Phase Peptide Synthesis (SPPS)

Fmoc-protected amino acids (AA) for SPPS were purchased from CHEM-IMPEX INT'L INC., Boc-Gly-OH was purchased from Novabiochem and *p*-SCN-Bn-NOTA was purchased from Macrocyclics. Analytical high performance liquid chromatography (HPLC) was carried out on a VWR Hitachi Chromaster system consisting of a Chromaster HPLC 5260 autosampler, a Chromaster 5160 pump, a Chromaster HPLC 5310 column oven and a Chromaster HPLC 5430 diode array at a wavelength of 215 nm). Elution of the samples was achieved using MQ water and ACN, both containing 0.1% TFA as eluent, using a standard gradient, ranging from 3% to 100% AcN over a 5.5 min time period with a flow rate of 3mL/min.
Peptides were purified by preparative reverse phase high-performance liquid chromatography (HPLC) on a Gilson HPLC system accommodated with Gilson 322 pumps over a Vydac 150H C18 column (10 µm, 250 mm x 22 mm) using a UV/Vis-156 detector at 215 nm. The solvent system consists of milliQ-water (containing 0.1% trifluoroacetic acid (TFA)) and acetonitrile (containing 0.1% TFA). A linear gradient was used starting from 4% of acetonitrile to 70% in 20 min at a flow rate of 20 mL/min.

Peptides were analyzed by electrospray ionization mass spectroscopy on a Micromass Q-Tof micro system coupled to a Waters Breeze analytical HPLC system equipped with Waters 2489 UV/visible detector (at a wavelength of 215 nm). The runs were performed on a Grace Vydac C18 column (15 cm x 2.1 mm, 3 µm) at a flow rate of 0.3 mL/min. The solvent system is constituted of water and acetonitrile (containing 0.1% of formic acid) and the linear gradient consisted of a 20 min run starting from 3% of acetonitrile to 100%. Electrospray data were acquired on Electrospray positive ionization mode scanning over the mass-to-charge ratio (m/z) scale from 100 to 2000. Data collection was done with Masslynx software.

1.2. GGGYK-NHCS-Bn-NOTA (Figure S1)

The *N*-terminally derived NOTA-peptide was synthesized using standard Fmoc strategy SPPS on Rink Amide resin (ChemImpex, polystyrene matrix, 100–200 mesh, 0.47 mmol.g^{-1}) in a fritted syringe reactor. AA (3 eq.) activation was performed with *O*-(benzotriazol-1-yl)-*N*,*N*,*N'*,*N'*-tetramethyluronium hexafluorophosphate (HBTU, 3 eq.) and *N*,*N*-diisopropylethylamine (DIPEA, 4 eq.) in dimethylformamide (DMF). AAs were coupled for 1h at room temperature (RT). Fmoc deprotection was performed using 4-methylpiperidine (20% (vol./vol.) solution in DMF), while standard washing steps were performed with DMF and dichloromethane (DCM). Alloc-protected side-chain lysine was deprotected in dry DCM (3 bed vol.) in presence of phenylsilane (24 eq.) and $Pd(PPh_3)_3$ (0.2 eq.). Residual Palladium was removed by washing the resin with 5 x 3 bed vol. of a solution of sodium diethyldithiocarbamate (2 mg/mL) + 0.01% (vol./vol.) DIPEA in DMF, 3 bed vol. of DMF, isopropanol, DCM and *p*-NCS-Bn-NOTA (1.4 eq.) in DCM/DMF (1:2) was coupled to the side chain of the lysine in presence of DIPEA (9 eq.) for at least 16h at RT. Cleavage from the resin was performed using a TFA/triisopropylsilane (TIS)/water (95:2.5:2.5 (vol./vol./vol.)) mixture at RT for 2h. After filtration and solvent evaporation under reduced pressure, the crude peptide was dissolved in water/ACN and lyophilized. After dissolution in dimethylsulfoxyde (DMSO) and filtration using a CHROMAFIL®

syringe filter. The collected pure fractions were combined and lyophilized to retrieve the purified peptide as a white powder with a purity > 95%, Mw(GGGYK-NHCS-Bn-NOTA) = 930.05 g/mol, Yield = 50%, [M+H+] 930.0309.

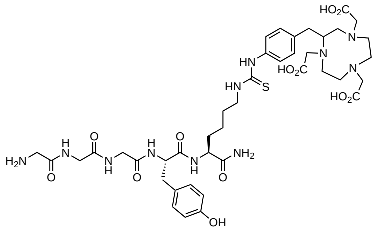

Figure S1. Structure of GGGYK-NHCS-Bn-NOTA

2. Quality controls of NOTA-Nanobodies (Nbs)

2.1. Size Exclusion Chromatography (SEC) analysis of the Nbs

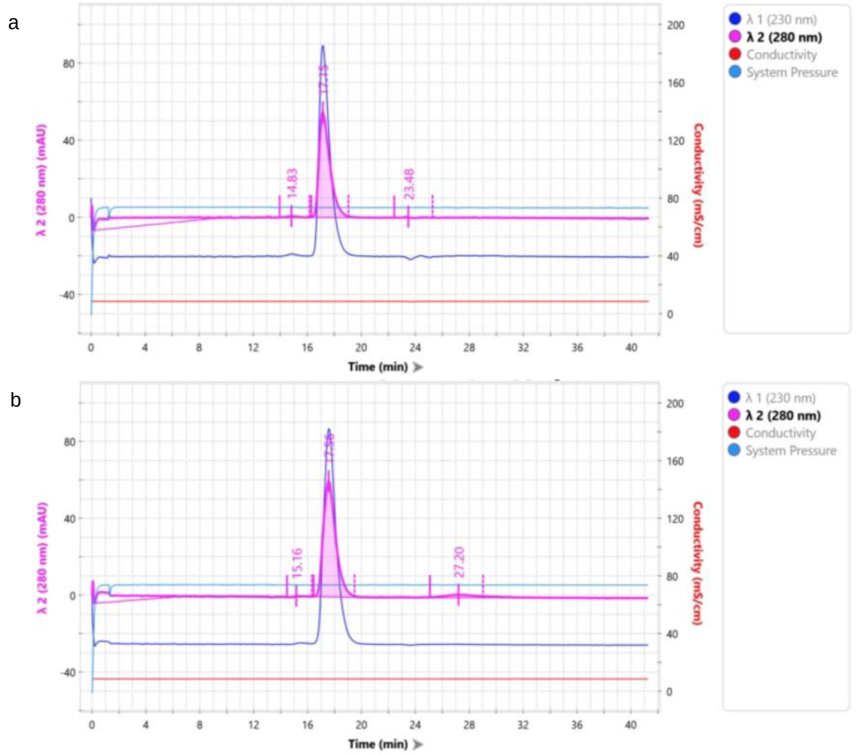

Figure S2. SEC analysis of the functionalized Nbs. (**a**) Site-specifically NOTA-coupled Nb, showing >95% purity (**b**) Randomly NOTA-coupled Nb, showing >93% purity.

2.2. SDS-PAGE and validation/quantification of Western Blot (WB)

The purity of the functionalized hPD-L1 Nbs was assessed SDS-PAGE and WB. Samples were prepared by diluting the Nbs in reducing buffer (Bio-Rad) and heating at 95°C for 4 min. 7.5 µg of protein was added to a NovexTM WedgeWellTM 8-16% Tris-Glycine gel (Live Technologies Europe BV). A sample containing unmodified Nb-His$_6$ was prepared as a positive control. SDS-PAGE gels were stained with Comassie blue. The gels were scanned on an Odyssey Infrared Imaging system (LiCor) using the following parameters: Preset: Proteingel; Resolution: 169 µm; Quality: medium; Focus offset: 1 mm; 700 nM; intensity: 6.0. For WB, transfer on the membrane (Bio-Rad) was performed at 70 - 100 V for 45 min – 1 h. The membrane was blocked with 1 g of Milk powder in 50 mL PBS, incubated at RT with the primary antibody (Mouse anti-His$_6$, 4E3D10H2/E3, dilution ThermoFisher Scientific) for 1h under gentle shake, then with the secondary antibody (Goat anti-mouse IgG HRP, Sigma Aldrich) following manufacturer recommended dilutions. The membrane was incubated for 20 min in the dark at RT with a freshly made HRP-revelation solution (18 mg chloronaphtol (Sigma Aldrich), in MeOH and 20 µL H_2O_2 (Sigma Aldrich) in TPA buffer pH 7.5 (0.5 M NaCl, 23 mM Trisma-base)). The membrane was analyzed visually.

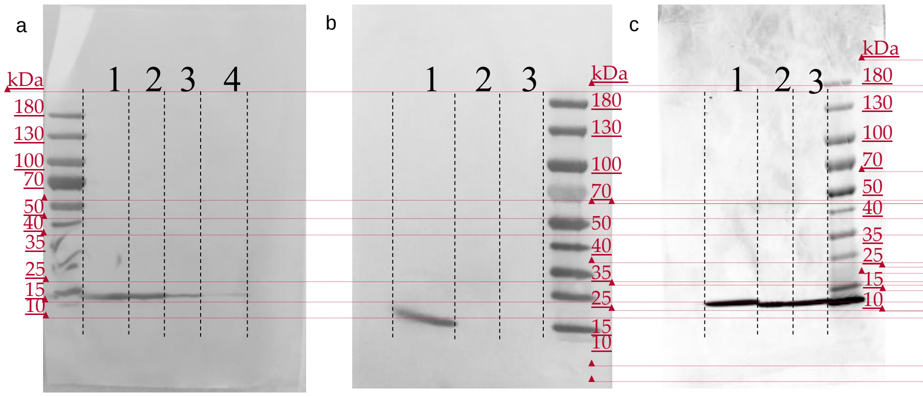

Figure S3. SDS-PAGE and WB analysis. Purity of site-specifically modified compound assessed by western blot with anti-His$_6$ staining, as compared with a ladder ranging from 180 to 10 kDa as represented on the side of each sub-figure -(**a**) Reference WB showing detectability of His6-tagged proteins using our WB experimental setup. Lane 1 to 4 contain decreasing amounts of His6-tagged Nb protein (5, 2, 1, and 0.5 µg respectively) showing that a faint band remains visible at the lowest mass. (**b**) WB of purified site-specifically modified NOTA-Nb. Lane 1 contains 7.5 µg of positive control (unmodified Nb), lane 2 and 3 contain 5 µg of purified site-specific NOTA-Nb from two different batches. No impurity by His$_6$-tagged starting materials is visualized. (**c**) SDS-PAGE corresponding to the WB from figure B confirming that protein is present at the expected Mw of the Nb in lanes 2 and 3.

2.3. ESI-Q-ToF

Assays were performed by the GIGA Proteomic Facility, Liège, Belgium. Samples were analyzed by ESI-Q-ToF at a protein concentration of 10 µM, 30% ACN, 0.5% Formic Acid (final) in ammonium acetate 25 mM.

a

b

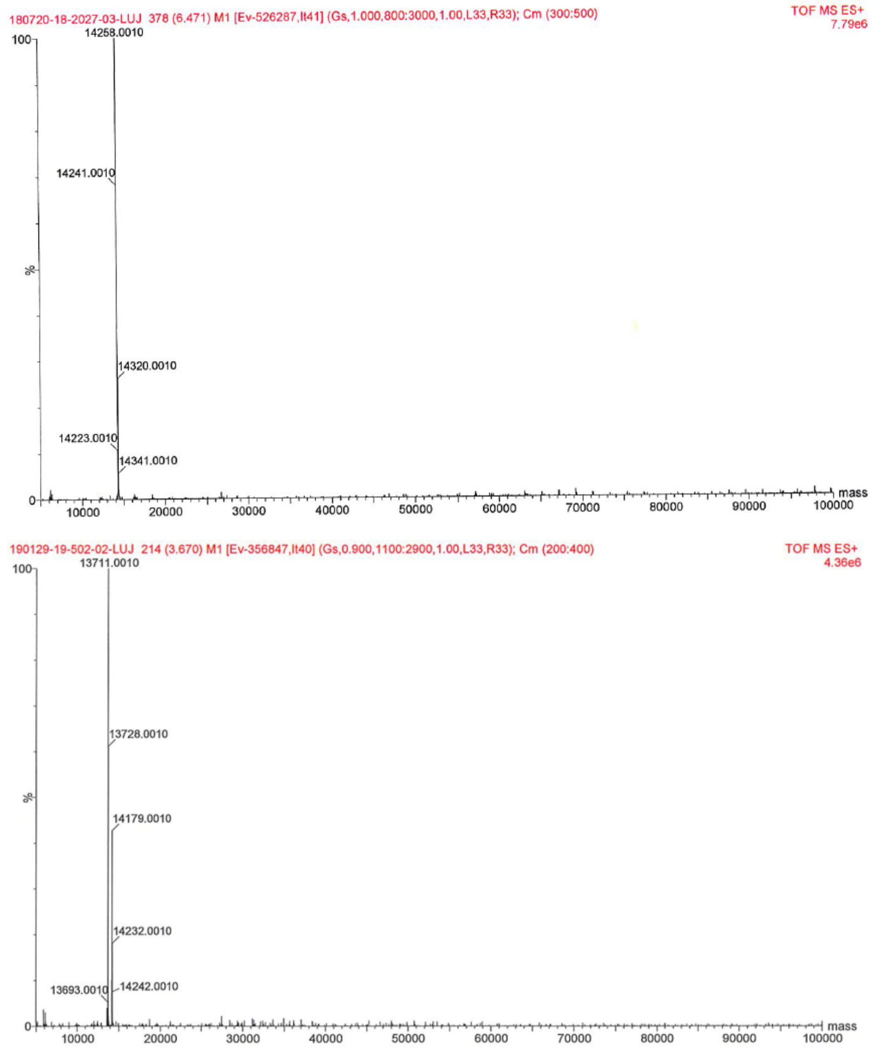

Figure S4. Mass determination analysis. (**a**) of the site-specifically modified NOTA-Nb showing major peaks of NOTA-Nb (14258 Da) and deamidated NOTA-Nb (14241 Da). (**b**) of the randomly modified NOTA-Nb showing major peaks of uncoupled deamidated Nb (13711 Da), uncoupled Nb (13728 Da), and coupled NOTA-Nb (14179 Da).

2.4. Surface Plasmon Resonance (SPR)

Measurements were performed on a Biacore T200 device (GE Healthcare) at 25°C and using Hepes-buffered saline (HBS; 0.01 M HEPES pH 7.4, 0.15 M NaCl, 3 mM EDTA, 0.005% Tween 20) as running buffer. The recombinant protein was dissolved to 10 µg/mL in 10 mM NaOAc pH 5.0 for immobilization on a CM5 sensor chip using linkage chemistry with 1-(3-(dimethylamino)propyl)-3-ethylcarbodiimide

(EDC) and *N*-hydroxy-succinimide (NHS). Unreacted EDC-NHS linkers were blocked with 1 M ethanolamine-HCl.

The modified Nbs were tested for affinity on immobilized human PD-L1 protein in SPR. To this end, 9 different Nb dilutions were allowed to bind to the target protein for 120 sec and dissociation was monitored for 160 sec. The equilibrium dissociation constant K_D was calculated by fitting the obtained sensor-grams to theoretical curves, assuming 1-to-1 binding geometries, using Biacore Evaluation software.

2.5. Cell binding study

The radiolabeled Nb binding capacity was tested on hPD-L1 positive (hPD-L1^{POS}) 624-MEL cells. 5x10^4 cells in 1 mL of medium per well were allowed to attach in a 24 well plate at 37°C two days prior to experiment. The plate was cooled to 4°C one hour prior to experiment. Supernatant was removed and cells were incubated for 1h at 4°C with 500 µL of a 3 nM or a 6 nM radiolabeled Nb solution in unsupplemented medium (N=3 wells per conditions). Unbound fractions were collected, wells were washed 2x with ice-cold PBS. Lysis of the cells was performed 2x with 0.75 mL of 1 M NaOH at RT for 5 min. All fractions were collected and counted in the γ-counter (Cobra Inspector 5003, Canberra, Packard). Specificity was assayed on hPD-L1 negative (hPD-L1^{NEG}) 624-MEL cells, and on hPD-L1^{POS} cells in presence of a 100-molar excess of unlabeled competitor (unmodified Nb) following the same procedures. Percentage of bound activity was calculated as followed: measured activity in bound fractions divided by the activity of the added solution.

3. *In vivo* stability studies

3.1. In vivo stability studies

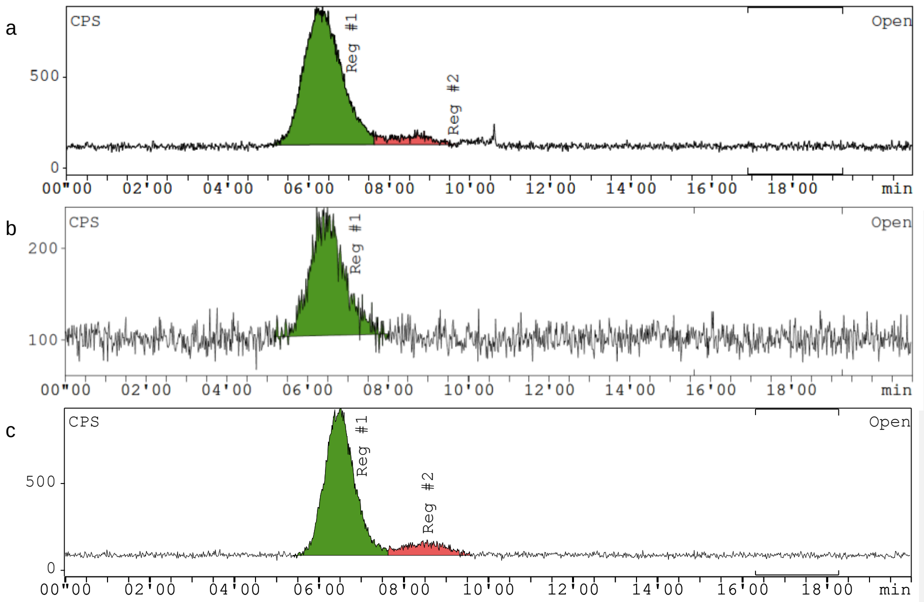

Figure S5. Radio-SEC analysis of metabolites for the site-specifically radiolabeled compound in blood and urine. (**a**) radio-SEC HPLC of the urine sample 2h post injection of the site-specifically labeled [^{67}Ga]Ga-NOTA-(hPD-L1) showing a % of intact probe of 93%. Rt(Nb) = 6.1 min, Rt(radiolysis) = 8.3 min. (**b**) radio-SEC HPLC of the blood sample 15 min post injection of the site-specifically labeled [^{67}Ga]Ga-NOTA-(hPD-L1). Rt(Nb) = 6.3 min. Blood analysis at later time points did not show any active compounds or could not be measured precisely due to low amounts of activity. (**c**) radio-SEC HPLC of the urine sample 2h post injection of the site-specifically labeled [^{68}Ga]Ga-NOTA-(hPD-L1) showing a % of intact probe of 89%. Rt(Nb) = 6.3 min, Rt(radiolysis) = 8.3 min.

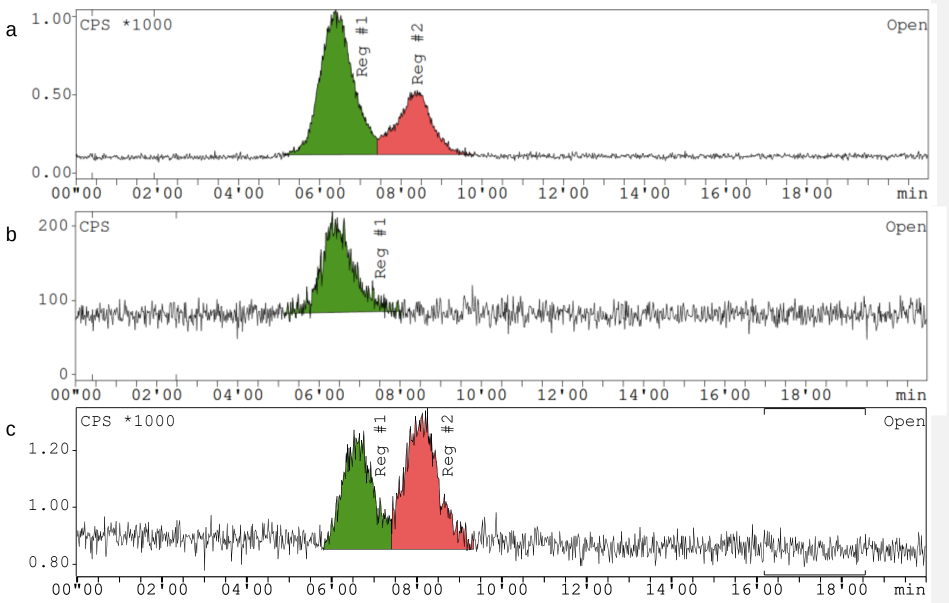

Figure S6. Radio-SEC analysis of metabolites for the randomly radiolabeled compound in blood and urine. (**a**) radio-SEC HPLC of the urine sample 2h post injection of the randomly labeled [^{67}Ga]Ga-NOTA-(hPD-L1) showing a % of intact probe of 69%. Rt(Nb) = 6.2 min, Rt(radiolysis) = 8.2 min. (**b**) radio-SEC HPLC of the blood sample 15 min post injection of the randomly labeled [^{67}Ga]Ga-NOTA-(hPD-L1). Rt(Nb) = 6.3 min. Blood analysis at later time points did not show any active compounds or could not be measured precisely due to low amounts of activity, showing that radiolysis product present in the urines is most likely induced by kidney metabolisation and is not re-absorbed in the blood stream. (**c**) radio-SEC HPLC of the urine sample 2h post injection of the randomly labeled [^{68}Ga]Ga-NOTA-(hPD-L1) showing a % of intact probe of 43%. Rt(Nb) = 6.4 min, Rt(radiolysis) = 8.1 min.

4. Biodistribution profiles

4.1. Biodistribution in C57BL/6 mice (N=6/group)

Table S1. *Ex vivo* biodistribution of the ^{68}Ga-labeled NOTA-Nbs in *C57BL/6 mice.*

	Site-specifically labeled [^{68}Ga]Ga-NOTA-(hPD-L1) Nb			Randomly labeled [^{68}Ga]Ga-NOTA-(hPD-L1) Nb		
	Mean (%IA/g)	SD	N	Mean(%IA/g)	SD	N
Blood	0.21	0.11	6	0.46	0.24	6
Heart	0.11	0.04	6	0.19	0.10	6
Lungs	0.38	0.05	6	0.42	0.10	6
Liver	0.67	0.16	6	0.51	0.09	6
Spleen	0.30	0.13	6	0.27	0.08	6
Pancreas	0.12	0.02	6	0.20	0.07	6
Left kidney	9.80	2.47	6	18.58	3.71	6
Right kidney	10.33	2.41	6	19.78	3.99	6
Stomach (without content)	0.17	0.07	6	0.32	0.15	6
Small intestine (without content)	0.44	0.62	6	0.31	0.12	6
Large intestine (without content)	0.14	0.04	6	0.22	0.09	6
White fat from pelvis	0.10	0.07	6	0.13	0.06	6
Muscle	0.07	0.03	6	0.12	0.06	6
Bone	0.11	0.03	6	0.24	0.06	6
Lymph nodes	0.14	0.05	6	0.25	0.13	6
Brown fat	0.08	0.03	6	0.17	0.13	6

Mean of %IA/g for each organ or tissue, with the standard deviation (SD) and number of sample (N) for both site-specifically and randomly labeled [^{68}Ga]Ga-NOTA-(hPD-L1) Nbs in C57BL/6 mice.

4.2. Preliminary data for statistical analysis: biodistribution and in vivo tumor targeting in athymic nude mice bearing hPD-L1^{POS} or hPD-L1^{NEG} tumors

Table S2. Preliminary data used for the statistical analysis: *biodistribution and in vivo tumor targeting in athymic nude mice bearing hPD-L1^{POS} or hPD-L1^{NEG} tumors.*

	Site-specific in PDL1^{NEG}			Site-specific in PDL1^{POS}			Random in PDL1^{NEG}			Random in PDL1^{POS}		
	Mean	SD	N	Mean	SD	N	Mean	SD	N	Mean	SD	N
Blood	0.26	0.23	6	0.15	0.10	12	0.30	0.21	6	0.07	0.01	6
Heart	0.14	0.11	6	0.09	0.06	12	0.16	0.10	6	0.04	0.01	6
Lungs	0.29	0.17	6	0.28	0.13	12	0.68	0.26	6	0.25	0.05	6
Liver	0.61	0.09	6	0.48	0.35	12	0.78	0.14	6	0.72	0.94	6
Spleen	0.28	0.06	6	0.22	0.16	12	0.34	0.07	6	0.09	0.03	6
Pancreas	0.10	0.05	6	0.08	0.05	12	0.12	0.05	6	0.06	0.05	6
Left kidney	6.69	2.45	6	6.67	2.77	12	17.43	2.64	6	13.46	2.33	6
Right kidney	6.73	2.26	6	7.60	1.78	12	17.19	2.90	6	14.41	2.40	6
Stomach (without content)	0.15	0.17	6	0.10	0.07	12	0.14	0.08	6	0.09	0.04	6
Small intestine (without content)	0.13	0.18	6	0.09	0.04	12	0.76	0.14	6	0.09	0.03	6
Large intestine (without content)	0.13	0.11	6	0.09	0.04	12	0.15	0.06	6	0.13	0.11	6
White fat from pelvis	0.09	0.09	6	0.04	0.03	12	0.09	0.05	6	0.08	0.01	6
Muscle	0.13	0.04	5	0.06	0.04	12	0.09	0.05	6	0.04	0.01	6
Bone	0.11	0.05	5	0.07	0.04	12	0.13	0.07	6	0.05	0.03	6
Lymph nodes	0.13	0.04	5	0.13	0.12	12	0.23	0.21	6	0.10	0.03	6
Brown fat	0.07	0.01	5	0.07	0.04	12	0.13	0.11	6	0.05	0.01	6
Tumor	0.49	0.36	6	3.22	1.20	12	0.33	0.12	6	2.11	0.80	6

Mean of %IA/g for each organ or tissue, with the standard deviation (SD) and number of sample (N) for both site-specifically and randomly labeled [^{68}Ga]Ga-NOTA-(hPD-L1) Nbs in both hPD-L1^{POS} and hPD-L1^{NEG} tumor bearing athymic nude mice.

Based on this data, Mean group 1 (site-specific) = 3.22 %IA/g (N=12), Mean group 2 (random) = 2.11 %IA/g (N=6), Stdev group 1 = 1.2, Stdev group 2 = 0.8, pooled stdev = 1.09087, and considering that 1% would be a relevant difference to observe in this model, to obtain 95% confidence we calculated using a Wilcoxon-Mann-Whitney analysis to determine that 21 animals per groups are necessary to conclude.

4.3. Comparative study: biodistribution and in vivo tumor targeting in athymic nude mice bearing hPD-L1^POS^.

Table S3. Comparative study of tumor uptake between the site-specifically and randomly ^{68}Ga-labeled NOTA-Nbs.

	Site-specifically labeled [^{68}Ga]Ga-NOTA-(hPD-L1) Nb			Randomly labeled [^{68}Ga]Ga-NOTA-(hPD-L1) Nb		
	Mean (%IA/g)	SD	N	Mean(%IA/g)	SD	N
Blood	0.36	0.15	21	0.34	0.07	21
Heart	0.14	0.06	21	0.13	0.02	21
Lungs	0.40	0.11	21	0.44	0.24	21
Liver	0.92	0.31	21	0.91	0.38	21
Spleen	0.43	0.24	21	0.44	0.19	21
Pancreas	0.10	0.04	21	0.10	0.02	21
Left kidney	8.44	2.66	21	13.81	2.57	21
Right kidney	8.02	1.15	21	13.77	2.85	21
Stomach (without content)	0.13	0.05	21	0.19	0.30	21
Small intestine (without content)	0.15	0.06	21	0.15	0.05	21
Large intestine (without content)	0.15	0.15	21	0.13	0.07	21
White fat from pelvis	0.09	0.04	21	0.08	0.04	21
Muscle	0.06	0.02	21	0.07	0.02	21
Bone	0.11	0.06	21	0.10	0.04	21
Lymph nodes	0.17	0.11	21	0.18	0.09	21
Brown fat	0.11	0.03	21	0.11	0.03	21
Tumor	1.89	0.40	21	1.77	0.29	21

Biodistribution and tumor uptake of the site-specifically and randomly labeled [^{68}Ga]Ga-NOTA-(hPD-L1) Nbs in hPD-L1^POS^ tumor bearing athymic nude mice (N=21/group, randomized), mean of %IA/g for each organ or tissue, with the standard deviation (SD) and number of sample (N).

4.4. Ex vivo analysis of the tumors by FACS

hPD-L1 expression on the cells from the dissected tumors was assessed. The dissected tumors stored in PBS (max. 12h) were cut, placed in 5 mL RPMI medium and treated using a gentleMACS™ dissociator. 150 µL of Collagenase from *Clostridium histolyticum* (Sigma Aldrich, 10.000 U/mL in PBS) and 150 µL of Dispase (Sigma Aldrich, 32 mg/mL in water) were added to the mixture and incubated at 37°C for 40 min. 2 µL of DNAse (1 mg/mL in PBS) was added to the mixture and treated 2 times on the gentleMACS™ dissociator. After filtration and centrifugation, red blood cell lysis buffer was added. The mixture was centrifuged, and the pellet was incubated with 100 µL of anti-mouse CD16/32 Antibody (clone 93, BioLegends, 1/200 dilution in PBS/BSA) for 10 min at RT. The pellets were incubated 30 min at 4°C with either 20 µL of isotype control solution (PE-CF594 Mouse IgG1, k Isotype Control, Clone X40 RUO, BD Horizon, 1.6/100 µL of PBS/BSA) or 20 µL of staining solution (PE-CF594 Mouse Anti-Human CD274, Clone MIH1 RUO, BD Horizon, 1.6/100 µL of PBS/BSA). Samples were resuspended in PBS/BSA for FACS reading (BD FACSCelesta™, BD Biosciences). % of cells expressing hPD-L1 is measured as the difference between the % of positive cells from the stained sample and the % of positive cells from the isotype control sample.

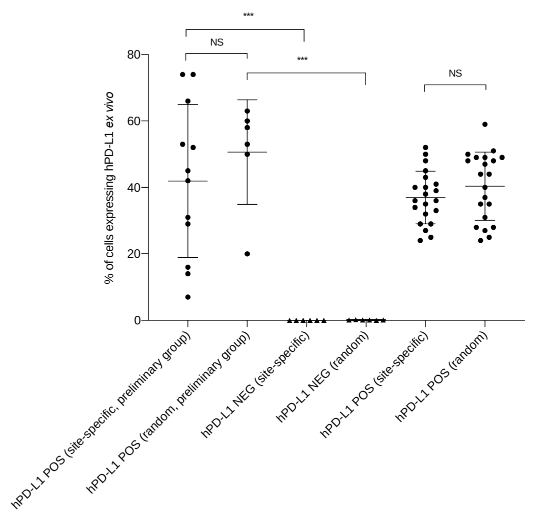

Formatted: Font: (Default) Palatino Linotype, 10 pt, Italic

Figure S7. *Ex vivo* assessment of hPD-L1 expression by FACS. hPD-L1 expression in the hPD-L1[POS] tumors after dissection of the animals (Difference NS between site-specific and random, p = 0.4173) (preliminary studies, two left lanes), expressed in % of cells expressing hPD-L1, as compared with the hPD-L1[NEG] tumors for both groups (P = 0.005 for site-specific groups, P < 0.001 for random groups) (two middle lanes). % of hPD-L1 expression on the dissected tumor cells for the comparison experiment (N=21 / group) is also presented (2 right lanes). Difference between the two group is NS (P = 0.2325).
